# Social Vulnerability and Mental Health Inequalities in the “Syndemic”: Call for Action

**DOI:** 10.3389/fpsyt.2022.894370

**Published:** 2022-05-30

**Authors:** Roberto Mezzina, Vandana Gopikumar, John Jenkins, Benedetto Saraceno, S. P. Sashidharan

**Affiliations:** ^1^World Federation for Mental Health, Woodbridge, VA, United States; ^2^The Banyan Academy of Leadership in Mental Health, Chennai, India; ^3^Madras School of Social Work, Chennai, India; ^4^International Mental Health Collaborating Network, Exeter, United Kingdom; ^5^Lisbon Institute of Global Mental Health, Lisbon, Portugal; ^6^Institute of Health and Wellbeing, University of Glasgow, Glasgow, United Kingdom

**Keywords:** community mental healthcare, mental health policy, COVID-19, mental health inequality, vulnerable groups, stigma, empowerment, Action Plan

## Abstract

Covid-19 is referred to as a “syndemic,” i.e., the consequences of the disease are exacerbated by social and economic disparity. Poor housing, unstable work conditions, caste, class, race and gender based inequities and low incomes have a profound effect on mental health and wellbeing. Such disparities are increasing between, among and within countries and are exacerbated by human rights violations, in institution and in society, stigma and discrimination. Social capital can mediate health outcomes, through trust and reciprocity, political participation, and by mental health service systems, which can be coercive or more open to demand of emancipation and freedom. Societal inequalities affect especially vulnerable groups, and Covid itself had a wider impact on the most socially vulnerable and marginalized populations, suffering for structural discrimination and violence. There are complex relations among these social processes and domains, and mental health inequalities and disparity. Participation and engagement of citizens and community organizations is now required in order to achieve a radical transformation in mental health. A Local and Global Action Plan has been launched recently, by a coalition of organizations representing people with lived experience of mental health care; who use services; family members, mental health professionals, policy makers and researchers, such as the International Mental Health Collaborating Network, the World Federation for Mental Health, the World Association for Psychosocial Rehabilitation, the Global Alliance of Mental Illness Advocacy Networks (GAMIAN), The Mental Health Resource Hub in Chennai, India, The Movement for Global Mental Health (MGMH) and others. The Action Plan addresses the need for fundamental change by focusing on social determinants and achieving equity in mental health care. Equally the need for the politics of wellbeing has to be embedded in a system that places mental health within development and social justice paradigm, enhancing core human capabilities and contrasting discriminatory practices. These targets are for people and organizations to adopt locally within their communities and services, and also to indicate possible innovative solutions to Politics. This global endeavor may represent an alternative to the global mental discourse inspired by the traditional biomedical model.

## Introduction: The Common Roots of Inequality, Vulnerability and Mental Health

The mental health impact of pandemic has been direct, a “crisis” response to trauma and fear of infection, resulting in anxiety, panic and depression. Public health measures, such as quarantine, lockdown and minor restrictions resulting in the disruption of social life, economic losses and individual lifestyles engendered feelings of uncertainty, helplessness and grief, made worse by lack of mental health services and supports (1). A decline in mental wellbeing has been clearly demonstrated among those who lost their job (2).

Covid 19 pandemic is so referred to as a “syndemic” (3) i.e., the consequences of the disease are determined by privilege and a variety of social and material factors and are exacerbated by social and economic disparity. This is “an illness of inequality” (4). Covid-19 does not discriminate, but it has a disproportionately negative impact on those most socially and economically deprived (5, 6).

This is similar to what happened after the economic depression in the 1920's, or the “Spanish flu.” In the “Bombay Influenza” there was exactly the same pattern: those most affected in India were caste hindus and those least Europeans; experience of loss of livelihoods and poverty steeply increased. The global emergency created by Covid-19 has confirmed that those affected most by the virus are the most vulnerable and most marginalized in our society, and this is also true for mental health. Their distress is often intergenerational, perpetuated by the shackles and legacy of structural discrimination and violence.

In this paper we address the interactions of mental health disparities and inequalities with population and individual vulnerability to higher risk of mental ill health during Covid-19 (7). Social factors are inherent to vulnerability, that increase the risk of mental health conditions; in turn, mental ill health can further engender social disadvantage and social exclusion.

Vulnerable groups are exposed to higher risk of illness and have limited personal and social resources to cope with its mental health consequences. Vulnerability to severe mental illness (8) is multifactorial and enhanced by the interaction of various factors e.g., biological-developmental, social, environmental, the exact mechanisms of which are largely unknown. We will focus on social vulnerabilities that are most amenable to interventions.

There is a significant body of scientific literature on the role of Social Determinants of Health (SDH) (9, 10) in the etiology and course of major mental disorders [(11, 12). Research has shown that higher rates of mental disorders are associated with social disadvantage, especially low income (13), limited education (14), occupational status (15) and financial strain (16), lack of social support (17), high demand or low control over work (18), critical life events (19), unemployment (20), adverse neighborhood characteristics (21)] and income inequality (22). These are identified as psychosocial risks of poor mental health. Understanding the relation between socioeconomic status (SES) and mental health depends on distinguishing its various measures and identifying independent associations with mental health conditions. Inverse relations between psychiatric disorders and socioeconomic status have been discussed in the literature according to the original dual hypotesis (23) of social causation–linked to adversity and stress–vs. social selection–downward mobility of genetically predisposed (24).

The link between poverty [either “absolute” or “relative”; cited by (25)] and mental health is bidirectional: poverty (lack of socioeconomic resources) increases the risk of exposure to traumatic experiences and stress that increase the vulnerability to mental disorders; while long term mental health problems can lead people into poverty due to discrimination in employment and reduced ability to work.

Adverse social conditions are associated with increased risk of specific mental disorders. Unemployment can increase the risk of common mental disorders, such as depression and anxiety (26), minority ethnic communities are more likely to experience mental ill health as a result of experiencing cumulative microaggressions including “structural” racism (27), stigma and socioeconomic deprivation (28). Gender inequality is linked to higher rates of mental ill health amongst women and creates barriers to accessing community mental health resources and care (29). Ecological risk factors for mental ill health include lack of adequate housing, transport, socially deprived neighborhood which are commonly associated with social disadvantage, as well as climate change and adverse environment (30). Being homeless or at risk of homelessness is strongly associated with mental health problems (31), as well as housing disadvantage (32). Societal segregation also has an adverse impact on mental health. For instance, people with learning disabilities, experiencing segregated schools and activities and who lack community connections are more vulnerable to hate, crime and discrimination, leading to increased risk of mental health problems (30). Urbanization and urban living are also risk factors for depression and anxiety. These are linked to socioeconomic deprivation and poor social support (33, 34).

According to the World Health Organization, health *inequalities* can be defined as “differences in health status or in the distribution of health determinants between different population groups” (35). Most inequalities reflect population differences in circumstances and behavior which are socially determined. Health *inequity* is defined as the absence of strategies to address unfair, avoidable, or remediable differences in health among population groups defined socially, economically, demographically, and geographically (36). To achieve health equity there should be fair distribution of health determinants, outcomes, and resources within and between segments of the population, regardless of social standing (37). This is a normative, aspirational concept, like the right to health. Health inequalities, as observable differences in health between subgroups of a population, are an indirect means to evaluate health inequities.

Health inequalities are closely related to social inequalities. Inequalities in health, and in health care, are patterned by a variety of socio-economic factors, income geography (for example, region or whether urban or rural), specific characteristics such as sex, ethnicity, disability, race, caste or socially excluded groups (for example, people experiencing homelessness). These disparities adversely affect groups of people who have systematically experienced greater social or economic obstacles to health based on their racial or ethnic group, religion, socioeconomic status, gender, mental health, cognitive, sensory, or physical disability, sexual orientation, geographic location, or other characteristics historically linked to discrimination or exclusion (36).

In mental health too, “systematic inequalities between social groups that are judged to be avoidable are inequitable and unfair.” This means that the disparities according to gender, age, ethnicity, income, education, and geographic area of residence are “inequitable and can be reduced by action on the social determinants.” (38).

Inequality and injustice are global challenges, including in high income countries with access to free education, health services and social security. For instance, in the UK life expectancy is 10 years shorter for people living in less deprived areas of the country (39), especially those from the black community. The social gradient in health has been defined as the graded relationship between social position and health, where health outcomes progressively improve with increasing social position (40). This is also true for mental health.

As noted by Desjarlais et al. (41), “exacerbating [social] conditions” act in *clusters*, in a circular interrelation with (often clustered too) “social pathologies” on one hand and “health problems” on the other hand. Another model is the one based on *negative spirals*, induced by risk factors interacting through multi-factorial models. As pinpointed by the World Health Report 20 years ago (42), there is a *vicious circle* of poverty (economic deprivation, low education, unemployment), mental disorders (higher prevalence, lack of care, more severe course) and economic impact (increased health expenditures, loss of job, reduced productivity).

## The Sources of Health Disparities and the Mental Health Care Gap

We will review some of the most important societal factors leading to mental health inequality, taking into account that action on social determinants of health and especially of mental health (38) would improve population health but may also increase inequalities in the distribution of determinants and, therefore, inequality of health outcomes (43). There is a need to understand how and why this is particularly true for vulnerable populations.

### Socioeconomic Inequalities and Geographical Distribution

There is a positive association between income inequality and mortality rates among countries within the Organization for Economic Cooperation and Development (OECD) (e.g., infant mortality rates among urban and rural subgroups) (44). All health indices are strongly influenced by income inequality, since they affect the standard of living (25). Such disparities are increasing between, among and within countries as confirmed in the first ever World Inequality Report (45). Health disparities have increased fastest in Russia, India and China. Europe is the least unequal region of the world, with a lower increase in inequality. Sub-Saharan Africa, Brazil and India, with the Middle East, are some of the most unequal regions. Inequity is increasing in countries as growth and development have not resulted in equitable standards of living.

One of the barriers to a fairer and better life for people in many countries is the problem of corruption, where money intended for individual poor people and those with mental health problems is stolen by politicians or other elites or diverted to criminal organizations. The relationship between corruption and inequality is bidirectional (46).

There are inequalities between different social groups in most countries and different levels of inequality among countries with a similar per capita income. Health and social problems (measured by index) are worse in more unequal countries (Portugal, UK, Italy, Greece vs. Scandinavian countries). Income inequality based on the top 10 percent income share has risen since 1980 in most world regions but at different rates: Europe 34 vs. Middle East 61% (47).

The empirical basis of the income inequality hypothesis (48) is now well-established for mental health although there is still a “fierce debate” on the nature of this association (49). Independently of parental social class, the risk of psychiatric disorder increases with increased downward social mobility and decreases with increased upward mobility (50). The lack of social mobility is linked to poor social and mental health outcomes as evidenced in many studies (51). Since social stratification is a critical component addressed in social mobilty, it can cluster with social capital.

It is also suggested that a “mixed neighborhood,” where people with low income can benefit from investment in social infrastructure, e.g., housing, ensured by the co-presence of those with higher SES, can have a beneficial impact (52). Income inequality may be more relevant in explaining health disparities in HIC than in LMIC (25), However, according to a recent systematic review, income inequality hypothesis as a driver of health inequalities is reported in the vast majority of studies (53).

Income alone is insufficient to assess inequality, and the Gini coefficient cannot measure inequality *per se*. We must also take into account socio-economic, political and cultural dimensions (54). Socioeconomic inequalities can be a cause of lack of social cohesion, but also a symptom of the same (25). The lack of social integration could explain the higher association between income inequality and psychosis, possibly related to experiences of paranoia (53).

In Europe, the burden of inequity explains the gap in health status between the poorest and richest income quintiles (47). The magnitude of this gap is attributed to 5 factors, controlling for age and gender (years 2003-2016):

- 35% income security and social protection.
- 29% living conditions.
- 19% social and human capital.
- 10% health service.
- 7% employment and working conditions.

### Social Capital

Social capital is a way of describing social relationships within societies or groups of people (17). This could mediate the association between income inequality and mental health. In the classic Putnam's definition, social capital includes community networks, civic engagement and participation; local civic identity encompassing sense of belonging, solidarity and equality; reciprocity and cooperation; and trust in others. According to Kawachi and Berkman (55), social trust is associated with “collective efficacy” at the “ecological” or local level in providing social support. Social capital can also influence access to community services and opportunities, and includes lobbying for their improvement or fighting against their cuts.

At a national level, social capital can foster civic and political participation. High measures of social capital in Europe accounted for 55% reduction in income inequality in Europe (49). Social capital influences and mediates health-related behaviors, through trust and reciprocity between citizens and the public domain and institutions (56). On the other hand, income inequality could affect social capital by eroding trust and cohesion of social networks. Feelings of trust and reciprocity are also important at the individual level (the s.c. “cognitive” social capital), promoting affective support, higher self-esteem and mutual respect (49). While there is strong evidence for an inverse association between cognitive social capital and common mental disorders, as well as significant evidence for group participation, the collective (ecological) measures of social capital do not confirm the same association (17).

Social capital, meant as resources in one's own social network (57), is a component of the set of “internal or external resources that can be drawn upon to initiate and sustain recovery” (58). The construct of individual's “recovery capital” encompasses economic, social, identity, personal and relationship capital, the significant gaps of which suggest a proactive capacity-building approach while working with the person and their social environment (59). The capabilities approach of Amartya Sen (60) suggests that a focus on capabilities or opportunities–rather than outcomes–will enhance individual agency.

Social capital is intrinsically linked to the resilience of individuals and communities and the two dimensions mutually influence each other. The response to Covid has determined interesting phenomena of resilience in low-income countries and there are studies, especially conducted in Africa, that show the connections between Covid, vulnerability and resilience (61–64).

### Social Vulnerability

Vulnerable populations differ from populations at risk, i.e., who have higher exposure to a specific risk factor. A vulnerable population is a subgroup or subpopulation who is “at higher risk of risks” because of common social contextual conditions that distinguish them from the rest of the population (65).

Disadvantaged, vulnerable and marginalized individuals and groups are defined by the WHO as those who, “due to factors usually considered outside their control, do not have the same opportunities as other, more fortunate groups in society.” There are many aspects of vulnerability, arising from various physical, social, economic, and environmental factors. Economic vulnerability, due to the impacts of hazards on economic assets and processes (i.e., business interruption, secondary effects such as increased poverty and job loss), is a form of social vulnerability, that is linked to the potential impacts of events on “special” groups (i.e., the poor, single parents households, pregnant or lactating women, people with disabilities, children, elderly).

Social vulnerability refers to the potential negative effects on communities of external stresses on human health, social mobility and social capital. Vulnerability is not just increased susceptibility to an illness, but also indicates reduced capability to develop resistance (and related measures to prevent loss) as well as resilience, the overall ability to recover. Nonetheless, coping strategies can be developed, meant as the ability of people, organizations and systems, using available skills and resources, to face and manage adverse conditions, emergencies or disasters (54). This is a dynamic process, not a static condition (WHO factsheet).

Many of these conditions that enhance vulnerabilities to health and, specifically, to mental health, with allied social risk factors, tend to define social groups and target populations, as we will see later on in the context of this “syndemic.” They are also related to social visibility and political recognition of those groups. We underline here that these are not just sociological but political concepts, because they can lead to health policies and care delivery, thus to the fulfillment of human rights.

### Human Rights, Empowerment, Stigma and Discrimination

Empowerment is a key to ensuring social justice (66). It is linked to the freedom of choice, i.e., the liberty to choose the kind of life that is worth living for anyone (67). This is a fundamental freedom, connected to human rights. Disempowerment, either at the individual or at the collective level, is linked to material conditions of life, but also to psychosocial (intended as the ability of people to control their own lives) and political factors, that is making their voices heard (25).

From the point of view of power differential, we cannot consider individuals and their health only in the context of simultaneous memberships in different status groups, as a sum of social positions or identities (68). “Intersectionality” is a theoretical framework that posits that social categories (e.g., race, ethnicity, gender, sexual orientation, socioeconomic status) intersect at the micro level of individual experience to reflect multiple interlocking systems of privilege and oppression at the macro, social-structural level (e.g. racism, classism, sexism) (69). They are referred to those social processes and can eventually result in what has been called “structural violence” by Galtung (1969) and others. A special effort should be made to assess inequalities in political power and cultural recognition especially for vulnerable population (54). This must result in ensuring civic, social and political participation that is a human right (art 19 CRPD).

When addressing inequalities related to human conditions and diversities, e.g., age, race and ethnicity, gender identity, disabilities, it is important to consider the pervasive stigma and discrimination against people with mental ill health and other socially excluded groups.

Stigma itself is “intersectional,” not just because it is related to social positions or social identities, but because it is linked to social processes of labeling and social exclusion (68). In turn, stigma and related discrimination often worsen inequalities experienced by socially excluded groups which further hasten their social exclusion.

Experiencing prejudice and discrimination can also compound and hinder recovery from a mental health condition, these amount to human rights violations, inside institutions as well as in the society.

There are inequities (due to inequalities) in ensuring the highest possible level of health (art. 25 of CRPD), through access to healthcare services and welfare provisions for social inclusion. The right to health and wellbeing is linked to social rights and access to social opportunities (again, to address the SDH).

### Access to Services and “Care Gap”

The abandonment of those in need of mental health care and treatment is a worldwide problem. This can be seen in the lack of welfare provisions by the state, availability of accessible and of affordable services, but also in the social exclusion and discrimination against people with mental health problems in both high-income and low/middle income countries. In most HIC there are also significant variations in access, experience and outcomes of mental health care sustained by social inequalities and vulnerabilities consequent upon economic, social, and cultural factors.

Recognition of mental health problems and diagnosis are strongly influenced by social status, and especially by belonging to a minority ethnic group. Even when accessing care, this is of poor quality and there are persistent risk of stigmatization, social exclusion, and loss of rights for these groups.

Mental health inequalities are made worse also by the use of coercion in mental health care. In Nordic European countries where welfare provisions are higher [see the (70)], social capital is high and inequalities less, there is greater use of coercion in mental health services compared to countries with higher inequalities, like Italy, Spain, or Brazil. This, while reinforcing stigma through involuntary placement and institutionalization, affects human rights and fundamental freedoms.

Although mental health is placed at the bottom of the political priorities, access to treatment and availability of appropriate care are important in addressing mental health disparities and inequalities. Nevertheless, these are two different concepts. Conventionally, “treatment” in mental health refers to psychiatric conditions, while “care” encompasses the whole range of psychosocial and community-based supports and interventions including individuals and their communities. “Care” must be holistic, or better, wholistic (71).

The mainstream approach to deal with mental health inequality is aimed to reduce the “treatment gap,” including the gap in access to social care (72). However, this current focus should be expanded to include social determinants of health, stigma, discrimination, fulfillment of human rights, and social inclusion. Moreover, not all social disadvantages can be pathologised or are linked with mental ill health. They persist as consequence of intergenerational injustices and structural barriers. Therefore, they cannot be viewed simply through a unidimensional mental health prism (73).

## The Higher Mental Health Impact of COVID on Vulnerable Populations

The WHO has issued guidance on actions required to address the mental health impact of the COVID-19 pandemic including the impact on service delivery systems (74). In the European Region (75), recommendations were framed across three key areas: general population and communities; vulnerable groups; and public mental health services.

Social inequalities influence mental health outcomes in vulnerable groups, who are considered at high risk. Vulnerable groups are at risk of differential care for the pandemic itself (prevention, PPI, appropriate care, vaccination), insufficient or not existing access to services they need and treatment gap, as well as continuity of care. Exclusion and the social gap result in a disconnection of a physical body from the “social body” (76). The risk of pandemic results in more self-isolation, prescribed physical distancing, and finally a more difficult restitution to the ‘social body'. Integration becomes more difficult when the social body is itself “sick.” Stigma can be “doubled” when people have Covid or affected by Covid.

### People With Pre-existing Mental Health Problems

They are some of the most vulnerable people to the contagion and may lack access to proper information and medical care. Their human rights, safety, protection, and even their environment, are at risk. More than ever, they could be forgotten, neglected and exposed to additional suffering because of the shortage of mental health services operating within communities, e.g., rehabilitation interventions, socialization activities and daycare centers, job placement, social enterprise, personal support, home and educational assistance services among others.

Psychiatric diagnosis by itself is a poor predictor of vulnerability to Covid. In identifying those at high risk, it is important to consider a range of social determinants of mental health and ill-mental health as well as subjective reactions based on individual life stories (77). People with severe mental illness who live in institutions, such as mental hospitals, nursing homes, halfway homes, social care homes, correctional facilities etc. are particularly at risk. These places have become more unsafe and provide less protection against the virus. Information asymmetry combined with poor adherence to public health protocols and overcrowding in these facilities results in sub-par care delivery. People living in institutions suffer from isolation and violations and, according to a recent WHO Europe survey of residential institutions, there were extensive violations of human rights in the first wave of pandemic (78). Life inside the institutions was difficult for both the residents and staff. The case for moving from institutions to community care has never been stronger.

### Marginalized Groups: The Social Gradient of Mental Health Is Reinforced

The poorest, those who are marginalized, confined in restricted spaces or living in unsafe and inhumane settings, as migrant and refugee population, especially those living in special centers and camps need psychosocial support and health protection help with primary needs. Homeless people living with mental illness are among the most vulnerable, lost in a social nothingness. Many of their natural support systems are no longer available. Traditional support networks and safety nets are also lost in isolation, and because of physical and social distancing under public health measures consequent upon covid. As a result, access to basic amenities and higher order needs such as that of affiliation are lost (79). Mental health services must increase outreach care for groups like this, and provide appropriate support to ensure their survival. This will involve mobilizing available resources of communities, volunteers, neighborhoods, associations and religious establishments.

### Wider Target Populations

There also other groups with higher levels of vulnerability, as the following.

(a) Older adults are more exposed to pandemic, and paid the highest cost, especially those living in residential facilities. They report higher depression scores for a number of and risk factors (isolation and detachment from society).

(b) Women have suffered the double burden of pre-existing inequities and the Pandemic. Reduction of employment has affected women disproportionately, and they also experienced severe burden in their career and educational role during Covid. Domestic violence is exacerbated by Covid. Globally, women are less likely to access health care, live poorer lives, experience loss of agency and control, be exposed to domestic abuse and intimate partner violence. Unemployment, alcoholism, closure of schools combined with the woman's social role as key care provider and family support have all been exacerbated by this stress.

(c) Health and social care front-line care workers (especially those working in Covid services), including mental healthcare workers are submitted to mental health distress and have also developed psychiatric disorders.

(d) Children and adolescents have suffered from school closure and distance learning (not available for all), lockdown in families, disruption of social life; feelings of isolation and loneliness, child adversities and violence within families, lack of schooling, unhealthy lifestyles all have a negative impact on mental health. Experience of relative poverty and access to technology; unemployment and closer experience of scarcity and interpersonal violence have all affected children from vulnerable communities.

(e) Other forms of physical and psychosocial disabilities can be equalized to the previous groups regarding social disadvantage in many ways. People with intellectual and cognitive disabilities do not know themselves how to avoid infection or its spread, as the information has not been interpreted in Easy to Read or verbally (80).

A first systematic review on the effect of studies health inequalitity factors on mental health outcomes during the pandemic (81) shows that risk factors are female sex, younger age, financial insecurity, having access to clear information about the pandemic, proximity to infection sites, pre-existing physical and psychological health conditions, stigma and discrimination because of one's identity (as belonging to an ethnic or sexual marginalized group). Studies were related to the first wave of pandemic and were mostly cross-sectional; only few of them were longitudinal or considering intersectionality among factors.

These assumptions must be confirmed by ongoing epidemiological surveys, longitudinal studies and long-term health surveillance (82). Recent data from the UK show that being a female, a carer, having an existing mental health diagnosis are independently associated with poorer mental health outcomes during the pandemic (83).

Vulnerability in children seems to be linked to special educational needs, acute or chronic diseases, living in single parent household or low-income families (84). During lockdown, resilience was associated with a positive appraisal style, mediated by perceived social support, and with the ability to recover from stress (85).

Migrants and asylum seekers have a 5 times higher prevalence of mental disorders (30). This is best explained by social factors that impact on mental health. Surveys show that mental health problems (anxiety, depression, stress and PTSD) are worsened as a result of unmet basic needs, social isolation and seclusion, lack of care and economic support, food insecurity, lack of safe and adequate shelter. Stigma and discrimination amplify and reinforce these socioeconomic disparities (86). A recent WHO survey, “Apart Together” (87), confirms the cumulative convergence of risk factors. A high proportion of respondents living in the most precarious settings (asylum centers, on the street and insecure accommodations), and experiencing daily stressors in relation to basic needs, reported deterioration in their mental health. They also reported an increase in discrimination since the beginning of pandemic, more in females and older people.

Similar findings are reported in other countries, for example in India (88), where large numbers of people migrate annually for employment in service, sales, building and domestic industries. They are exposed to discrimination, work-rights exploitation and job insecurity and these have worsened during COVID-19 with increasing concerns over mental health, especially among those living in migrant camps.

## A Map of Interacting Domains

Health inequities are modifiable. They are a product of structural and political processes that affect the everyday living conditions of individuals and populations; they are socially determined, driven by the conditions of daily life, in power, money and resources. Therefore, any action on health inequities requires, first of all, an action across all the social determinants of health, i.e., the range of interacting factors that shape health and wellbeing. “Equity stratifiers” (36), reflect social conditions (socioeconomic status, education, place of residence, race or ethnicity, occupation, gender, religion) and intersect, e.g., education increases health literacy, as information, decisions, following treatment. Reducing health inequities is an ethical imperative, because “social injustice is killing people on a grand scale” (89). Comprehensive strategies are needed to address societal determinants in order to improve mental health in the population and reduce inequities (38).

Anyway, as we have seen, this is not simple and has to deal with many domains, whose conceptualization, starting from the wide and variable list of SDH (43), is often differently defined. Areas of ambiguity are also due to the fact that the same phenomena or social categorizations belong simultaneously to one or more domains, and therefore intervene in a different way according to the sphere or domain under analysis. Finally, these domains largely overlap each other.

**Figure 1:**
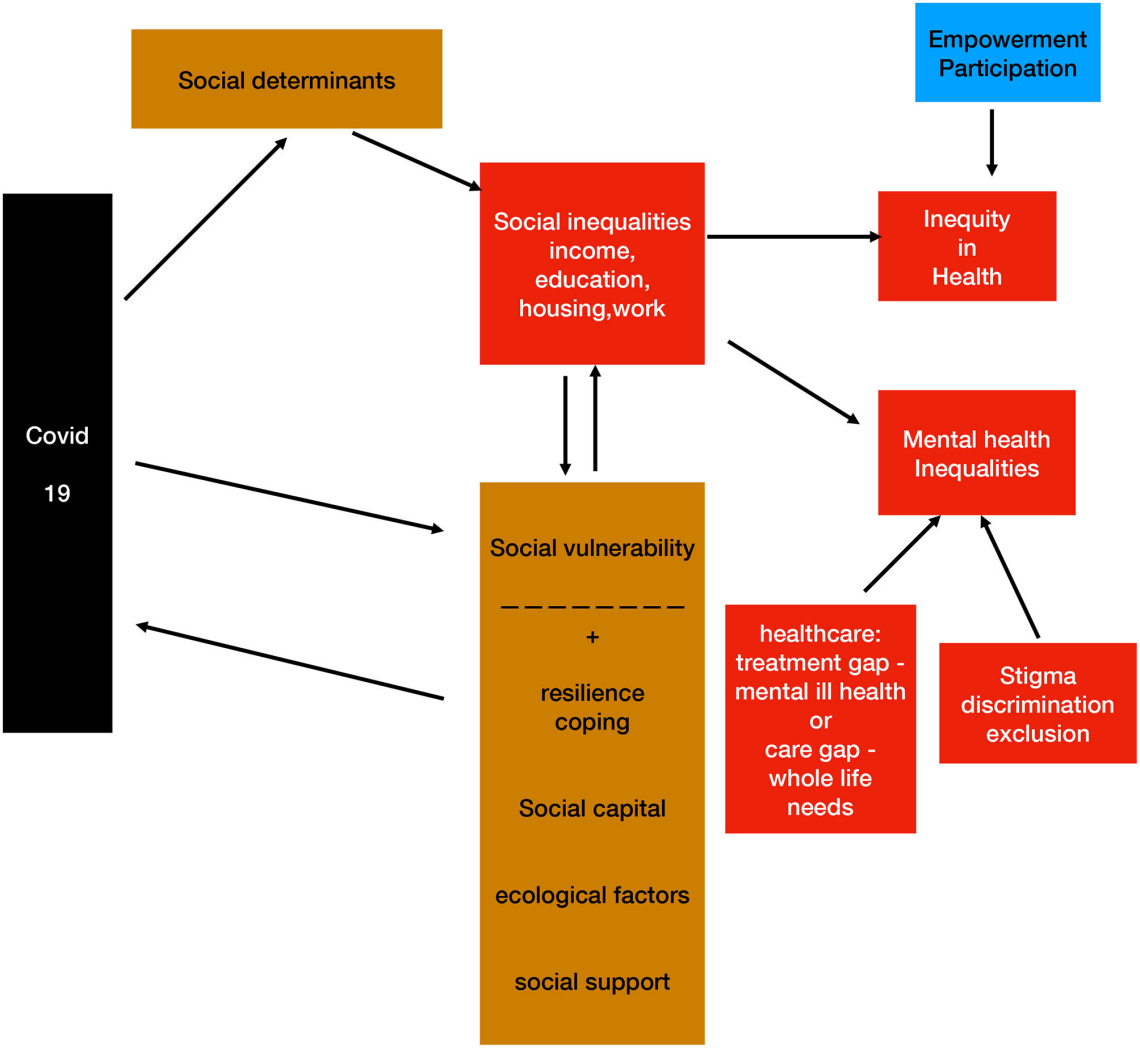
A map of interacting domains and concepts.

The complex relations, interactions and associations among these domains, and their impact on mental health, are outlined here in a flow-chart. This is intended as a descriptive map rather than a theoretical model or framework [see (90)], which would require further empirical and scientific evidence. Due to the multifactorial complex nature of causality in health, there is a web of determinants that creates a form of non-linear, complex system (91). Clustering methods (92) may help us understand this better, especially if “person-centered” (68), in order to catch the complex interactions of these cathegories - or “equity stratifiers”–on individual health and wellbeing. However, transformative action is therefore necessary and cannot be guided by theory alone, but must address emerging needs. Thus, we claim the link between theory and practice and the heuristic value of the knowledge that is acquired empirically in the processes of transformation: not just evidence-based practices, but practice-based evidence (see Figure 1).

Social determinants, through differential distribution, affect social inequalities (e.g., economic status, education, housing, work, etc), and thus can create inequity. These have an impact on mental health inequality, which is influenced by access, quality and outcome of treatment of mental ill health conditions but also by overall comprehensive care (if focused on whole life needs).

Stigma, discrimination and social exclusion also increase mental health inequality in many ways we outlined. Covid 19 impacts directly on SDH, with a gradient that is affected by a number of mediators / modulators, with a potentially negative (higher social vulnerability, that is also linked to SDH) or positive effect (resilience, coping, social capital, ecological factors and social support regarding both communities and individuals). Empowerment, especially as far as civic, social and political participation is concerned, can potentially contrast inequity and promote social justice.

## Action Required

### Policy Level

Action requires a set of conditions, such as strong national governance, public participation in policy-making, health sector orientation, and strong global leadership, always maintaining the focus on improving health equity (38).

We argue that while *equitable health* access and interventions are the key, we recognize the need for politics of wellbeing to be embedded in a system that places mental health within the development space and within a *social justice* paradigm (93). This will open up potential responses, both biomedical but also aimed at enhancing core human capabilities and challenging discriminatory practices.

*Addressing social determinants* is accepted as a priority but nothing much has happened despite research, evidence and policy. We must take into consideration the social gradient of mental health, the recognition of inequality, more than that of basic needs. As described before, Covid-19 has shown that homeless people are moving in an increasing social void. Similarly, there is a challenge of welcoming migrants and integrating them at the same time when there is a crisis of our societies.

*Focus on human resources* is required and it is connected to enhancing the status and role of social work and development practitioners in mental health. If social determinants are to be adequately addressed, mental health professionals have to embrace diversity in the workforce and welcome the wisdom, skills and expertise of social work practitioners such that the goals of poverty reduction, gender parity, access to housing, mitigation of stigma, formation of support circles, catalyzing social change by building equity through facilitation of employment and cash transfers are advanced.

*Focus on appropriate, humane care must inspire community inclusion*. In the event that treatment options are to be made available to address emergency and acute needs for vulnerable persons such as homeless individuals, in mental health care services, the discussions must transition from binary positions to the “how's” and the “when's” and focus on the social architecture of such spaces, reframing care paradigms and using spaces therapeutically, e.g., by using peer led collaborative social audits.

Emerging evidence suggests that *increasing social cohesion and social capital* may reduce the negative effects of neighborhood deprivation on mental health (30). The “whole society approach” (94) suggests that new forms of social connection should be developed and enhanced as part of a collective effort to tackle the social problems thrown up by Covid-19. It has never been more urgent to step aside from individualistic notions of health and embrace the values and practices of sharing and solidarity, both civil and social. This will enhance our sense of being part of a “more resilient”community and empower individuals and communities. This whole society task is either a service imperative or a political priority.

We need to avoid fragmentation of our efforts by building *alliances between public mental healthcare, social services and third sector*. This will ensure an effective response to whole life needs, protecting not only health but also human rights of people living in institutions, hospitals, prisons, shelters, nursing homes, group homes and other special facilities, and those experiencing social deprivation.

It is important to implement the *national emergency plans* dedicated to mental health in all affected countries, and related preparedness, as called for the World Health Organization (72) and the UN (95). An appeal has been launched by the World Federation for Mental Health (80).

### Service Level

Many Mental Health Service Organizations are recognizing the need to transform the mental health system. Covid−19 has highlighted the urgent need to act now. This transformation requires a fundamental change in thinking about mental health through a comprehensive review of current services and practices (96). Community mental health services, which have a long history of community networking and engagement, can act as exemplars and provide essential bridges to a “new normal,” with collaboration and shared responsibilities at its heart. Community-based care must include more information, greater community involvement and responsibility rather than a paternalistic top-down, often authoritarian approach, and eventually more reciprocity within the inclusion process. We need a *rights-based and person-centered, but also a whole community appr*oach (96).

As important as the restoration of rights is, Basaglia's notion of freedom (1987) goes beyond a narrow concern with legal status. It is similar to that offered by Amartya Sen (1999) that freedom is the foundation, rather than the result, of human and economic development. In much the same way, Basaglia's thought of freedom as a prerequisite for effective care, rather than a condition for which one strives through treatment. This notion of freedom as a key cross-cutting dimension should be driving any mental health service transformation.

Stemming from “*Big Issues identified by Covid-19 requiring Fundamental Change in Mental Health*,” a Local and Global Action Plan has been launched by a coalition of organizations representing people with lived experience of mental health care; who use services; family members, mental health professionals, policy makers and researchers, such as the International Mental Health Collaborating Network, the World Federation for Mental Health, the World Association for Psychosocial Rehabilitation, the Global Alliance of Mental Illness Advocacy Networks (GAMIAN), The Mental Health Resource Hub in Chennai, India and others (96). All these organizations are united in this global endeavor and offer an alternative to the global mental discourse promoted by academic and (mostly) western institutions inspired by the traditional biomedical model. The dominant clinical response today does not acknowledge or address the importance of social determinants in the current service model. The consequence of this is that life circumstances such as, poverty, inequalities, systemic racism and discrimination are not systematically addressed, while there is clear evidence that social determinants play a fundamental part in the lives of people with mental health issues.

The targets of the Action Plan are for people and organizations to use locally within their communities and mental health services (see Table 1).

**Table 1:** Big issues and actions for change: an international and local action plan 2020−2030.

**Big issues**	**Actions for change**	**Action points: examples**
1 Society and Responsibility	Building societal responsibilities and responses to people with mental health issues	A society should take responsibility for determining and meeting the mental health and well being of its citizens founded on human and civil rights and a collective vision of responding to peoples' social and economic needs
2 Coping and resilience	Learning how service users and families have coped during the Pandemic. Discovering and valuing the ways people have shown their resilience and ingenuity at this time.	Gathering evidence of innovative practices and approaches that provided support during the pandemic. These activities must be captured, through action research and open discussion forums with service users, family members, peer workers and organisations
3 Action on social determinants	There is a fundamental need to focus on understanding the importance of the social determinants of mental health in meeting the whole-life needs of people	Developing local strategic plans on the social determinants of mental health through a community partnership that acknowledges international frameworks and goals
4 Changing the thinking	Changing the thinking within mental health services, professionals, organisations and communities	Promoting a different belief that people can recover and discover a life of hope and purpose, especially amongst the professionals
5 Connection and involvement	Increasing the ways to keep people connected, involved, informed and supported	Bringing the voice of service users and families into the centre of decision making by ensuring that there is real connection, trust and support
6 Human Rights	Acting on Human Rights and Fundamental Freedoms: Moving toward avoiding coercive practices	Reducing the emphasis on risk aversion. Avoiding involuntary treatment orders, locked doors, seclusion and restraint. Changing attitudes, embedding evidence-based approaches to reduce and eliminate these forms of control
7 Education and Training	Developing education, training, continuing education and retraining of all mental health workers to increase our skills and knowledge on recovery, social inclusion and community partnerships	Enlisting universities and colleges and professional organisations to review foundation and continuing training curriculums toward developing new learning, based on human rights and recovery and discovery approaches
8 Self Help	Ensuring knowledge and availability of existing person centred self-help, tools and instruments; increasing the use of psycho-educational resources; and developing new resources	Ensuring the availability of existing person centred materials, books, apps, tools and instruments.
9 Mutual Support	Establishing and promoting mutual support for service users and carers with the aim to enhance togetherness, knowledge, resilience and to increase hope and sense of belonging	Developing mutual support groups, peer led groups, self help groups, community forums, citizen assemblies, formal and informal advocacy
10 De-institutionalisation	Closing the large psychiatric institutions through strategic and incremental plans, simultaneously increasing the availability of comprehensive community mental health service systems	Relaunching the campaign to close all psychiatric institutions by using all forms of social media, webinars, online conferences, seminars, tutorials and forums
11 Discovery and Recovery	Increasing the availability and choice of discovery and recovery informed practices.	Identifying and making available the wide range of discovery and recovery informed practices, tools and therapies
12 Life expectancy	People diagnosed with mental health conditions must have increased life expectancy and this issue must be urgently addressed	Mental health organisations must focus on increasing the life expectancy of people with mental health problems and issues

Among the 12 “Big Iussues and Actions for Change” there is the recognition of the “fundamental need to focus on understanding the importance of the social determinants of mental health in meeting the whole–life needs of people.” Recommended action points are:

- Developing local strategic plans on the social determinants of mental health through a community partnership that acknowledges international frameworks and goals.
- Mental health providers must prioritize these local strategic plans as they are of equal importance to the development of clinical services.
- Applying a co-production methodology: A democratic and inclusive process of development must encompass all local stakeholders as equal partners to create a *Whole Life - Whole System* approach.
- Working with Non-Governmental Organizations and a range of different agencies (public and private) that provide significant services in our societies. We especially need to work with them to meet the whole life needs of people in the community.
- There is a need to increase and sustain the funding of community organizations that provide essential services not met by statutory organizations.
- Addressing inequalities, systemic racism and discrimination. Ensuring that the needs and voices of oppressed, marginalized and vulnerable groups are prioritized, and this injustice is addressed through specific actions by applying equalities principles.

The various practical actions required to address social determinants in relation to mental health, at various levels, are summarized here:

#### Global Level

i. *Commitment and consensus* by international bodies and professional organizations on the need to address social determinants of mental health.
ii. Addressing social determinants is accepted as a *priority*.
iii. Social determinants of health (including mental health) as part of global *discourse on reducing health inequities*.
iv. Global leadership to address health inequities.

#### National Level

i. Include *strategies and plans* to reduce health inequities as national policies on mental health.
ii. Ensure mental health policies are linked to broader *social justice* agenda.
iii. National *leadership* to reduce health inequities.
iv. *Focus on human resources*–enhancing the status and role of social work and deployment of community development resources as part of mental health.

#### Regional Level

i. Close alignment of public (population) health and mental health priorities.
ii. Local plans to integrate social inclusion practices as part of mental health–focus on socially excluded populations, for e.g., homelss, refugees and minority ethnic groups.
iii. Comprehensive data on health inequities (morbidity, access, experience and outcomes) and annual appraisal of progress.
iv. Plans to develop resilient and inclusive communities–specific actions based on local needs and priorities.
v. Greater investment in community, voluntary groups, to develop community assets to reduce social exclusion and marginalization.

#### Service Level

i. Ensure that all mental health services are *rights-based and person-centered*, and adopt *a whole system / community approach*.
ii. Review and reduce all practices that restrict individual rights and freedoms.
iii. Provide training and support for mental health workforce to improve their capability and capacity to understand and reduce health inequalities and inequities.
iv. Systematic audits of mental health services in relation to reducing mental health inequities in the population they serve.

### Some Examples

a. The implementation of accessible financial services is essential to tackling poverty, empowering people (particularly women) and communities (36). Poverty alleviation tools, including cash transfer, show a significant positive effect on mental health of adult population. There is evidence of positive effects of Unconditioned Cash Transfer Programmes on Mental Health Outcomes in African countries, e.g., Kenya and Uganda and others (97, 98).

b. In India, the needs of homeless persons and homeless persons with mental illness (HPMI) have been recognized as critical to promote equitable development and services. Homeless persons and people who migrate from one region or state to another are amongst those worst affected by the pandemic, owing to housing and work instability and experiences of ill health and scarcity. Many homeless persons who had migrated in search of better livelihoods, especially employed in the informal workforce or seeking better options for their health concerns, suffered tremendous losses owing to the multiple lockdowns and the lack of support nets. Consequently, the National Urban Livelihoods Mission (NULM) supported shelters have been scaled up to support homeless persons in distress across many states; demand however outweighs the supply chain. The Government of Tamil Nadu, meanwhile, are in the process of developing a Statewide Policy that addresses the needs of those experiencing homeless and mental ill health. In addition, they have scaled up services for HPMI, in district hospitals adhering to international human rights tenets that promote integrated and person- centered care. Many states have also adopted inclusive living approaches such as “*Home Again”* that provide community living options for persons with long term mental health needs, ensuring that participation and agency are advanced (99, 100). Legal activism has also supported these changes in care paradigms. Intersectoral collaboration, recommended by the Mental Health Care Act (MHCA, 2017), the Rights of Persons with Disabilities Act (RPDA) and in keeping with the vision and spirit of the first Indian Mental Health Policy, also addresses the social determinants of mental ill health by using a “whole body, whole life approach” that focuses on poverty reduction, livelihood promotion and access to nutrition and other social security schemes. This process is in its early stage; a much-needed next step is to ensure consistent service delivery and transformative shifts at systemic levels.

c. The European Union recently decided to foster and complement national plans via the largest-ever stimulus package of €1.8 Trillion to build a greener, more-digital and more-resilient post-COVID-19 Europe. The plan is called “Next generation EU” (101). A pre-existing “Social Economy” includes cooperatives, mutual societies, non-profit associations, foundations and social enterprises, which operate a very broad number of commercial activities, provide a wide range of products and services across the European single market and generate millions of jobs. According to the EU Commission, there are 2 million social economy enterprises in Europe (10% of all businesses in the EU, more than 11 million people–about 6% of the EU's employees). Their members act in accordance with the principle of solidarity and mutuality and manage their enterprise on the basis of 'one man one vote' principle. It substantially contributes to economic, social and human development across and beyond Europe and also supplement existing welfare regimes in many member states. It addresses several key EU objectives: sustainable and inclusive growth, employment, social cohesion, social innovation, local and regional development and environmental protection, but also individual's wellbeing (102).

d. In Italy, part of these new “recovery funds” will be aimed to digital transition, green economy and also at strengthening social cohesion by reducing social inequalities. This should create work opportunities, through a welfare community with the involvement of social economy and social enterprises. It is still unclear if there will be any dedicated investment in community mental health services, or to welfare services with an impact on the living conditions of people with mental health issues, at risk of marginalization and social exclusion. Mental health services in some regions have developed differentiated strategies with the help of NGOs such as social cooperatives and voluntary associations. Based on the principles of social and health integration, these policies and programs provide for integrated social and health paths, basic social support, the right to independent living, training and job placement. One of the main organizational-strategic aims is the construction of “personal budgets,” in which the suffering individual has an active role and a bargaining power. They provide the person with support in the exercise of fundamental rights and in accessing social opportunities (home, education, training at work, health management, leisure activities), and for capacity-building paths in relationship with other services and institutions, toward a higher autonomy. This shows that social determinants can be directly addressed, both at the individual level and at the level of socially vulnerable groups.

e. Health Care Workers (HCWs) have an important role in addressing social determinants as part of mental health care and treatment. This has been highlighted in the context of dealing with the consequences of the recent covid pandemic and restrcitions as part of “lockdowns.” HCWs are considerd as one of the most affected groups and specific strategies are required to support and protect them from the health (including mental health) consequences of the syndemic nature of this pandemic (103).

## Conclusion

Health inequities amount to major social injustice in modern societies. Systematic differences in health between groups of people are unfair and avoidable. They are created and sustained by structural and political processes that affect the everyday living conditions of individuals and populations (36).

With covid-19, mental health professionals are asked to contribute with an unprecedent effort to deal with the highest and sudden increase of mental distress in society since last century, as well as to help especially those with severe mental issues to receive care and support.

In this context, it's time to recognize the specific impact of mental health inequalities worldwide, especially after this “syndemic,” when disparities are also increasing between and among countries (104). We know that poor public service provision and limited or no access to healthcare, lack of housing and work policies and practices, result in poor health and damaging social outcomes. As we have described, Covid-19 has enhanced social, health and mental health inequality, with related violations of human rights, increase in stigma and discrimination (105).

Looking that healthcare contributes to mental health disparities and inequalities in minor part, we should abandon the reductionist perspective that mental health inequalities are mostly related to access to treatment. They stem from social inequalities and are worsened by vulnerability conditions.

Inequalities are part of health as well as economic and social field; they continuously interact with stigma and discrimination.

Not just mental health must be included in all policies, but also all policies must be considered for providing a whole life approach and a new paradigm of mental healthcare.

Stigma hinders multi-sectoral collaboration and cooperation, as the access to social services and welfare. Thus, a robust antistigma campaign for universal access is highly needed.

Inequality concerns access not only to care, but to all social opportunities and inclusion, in order to overcome stigma and discrimination, and it cannot be realized without a wider, integrated political effort. It is a political issue as well as a civic imperative for all of us.

Addressing social determinants of health, to ensure “social” rights and achieve social justice, is a huge task. This is linked to empowerment and participation of stakeholders, especially those belonging to vulnerable groups, and stemming from those using services and caregivers. This calls for collaborative plans and work by international organizations, mental health activists, workers, users, carers and political leaders of change.

## Author Contributions

RM and SS developed the conceptual framework and the overall organization of the paper. RM also worked on literature review, policy papers, and conceptual mapping. SS worked on the overall re-organization and revision of the manuscript. VG contributed to a global mental health perspective and through practices and policies on vulnerable and marginalized groups. JJ is the main author of the action plan and its conception. BS contributed especially to social determinants, global mental health, human rights, and public health. All authors contributed to the article and approved the submitted version.

## Conflict of Interest

The authors declare that the research was conducted in the absence of any commercial or financial relationships that could be construed as a potential conflict of interest.

## Publisher's Note

All claims expressed in this article are solely those of the authors and do not necessarily represent those of their affiliated organizations, or those of the publisher, the editors and the reviewers. Any product that may be evaluated in this article, or claim that may be made by its manufacturer, is not guaranteed or endorsed by the publisher.
